# Effects of repeated culture in sub-inhibitory concentrations of ciprofloxacin on resistance and genetic characteristics of an ocular *Pseudomonas aeruginosa* isolate

**DOI:** 10.1007/s11274-026-05105-7

**Published:** 2026-06-29

**Authors:** Tanzina Akter, Mahjabeen Khan, Fiona Stapleton, Mark Willcox

**Affiliations:** 1 School of Optometry and Vision Science, Faculty of Medicine and Health, University of New South Wales (UNSW), Sydney, NSW 2052 Australia; 2 Microbial Biotechnology Division, National Institute of Biotechnology (NIB), Dhaka, 1349 Bangladesh

**Keywords:** *Pseudomonas aeruginosa*, Ciprofloxacin, Resistance, Mutations, Sub-MIC, *gyrA* and *mexS*

## Abstract

Purpose: To analyse the resistance and genetic changes of a sensitive ocular isolate of *Pseudomonas* *aeruginosa *to repeated exposure to subinhibitory concentrations of ciprofloxacin. Methods: A ciprofloxacin-sensitive ocular isolate *P. aeruginosa* PA123 was serially passaged under gradually increasing sub-inhibitory concentrations of ciprofloxacin for 18 passages. The minimum inhibitory concentration (MIC) was determined daily using the broth microdilution method, and after 18 passages when resistance had plateaued, ciprofloxacin was withdrawn and passaging continued for a further 13 days. A control culture in the absence of ciprofloxacin was passaged sequentially for 31 days. Genomic DNA from selected passages was extracted and subjected to whole-genome sequencing. Sequencing reads were quality-trimmed using Trimmomatic 0.38 and then analysed with Snippy 4.6.0 to identify acquired mutations using parent PA123 (non-passaged) as a reference strain. Results: The parent strain had an initial MIC of 1 µg/mL, this increased during growth in sub-MIC ciprofloxacin two-fold at passage 4 (2 µg/mL), then progressively increased to 64 µg/mL at passage 15, at which it remained for a further three passages. Upon removal of ciprofloxacin, there was no change of the MIC (64 µg/mL) during the subsequent 13 passages. In early passages (P1-P7), PA123 acquired a Leu240 frameshift mutation in *mexS*, while in intermediate and later passages, Thr83Ile, Ala51Val, Asp87Tyr, duplication of alanine at position 570 (Ala570dup) mutations occurred in *gyrA* gene. Isolates from control passages acquired only background mutations unrelated to ciprofloxacin resistance. Conclusion: Stepwise accumulation of mutations in *mexS* and *gyrA* under sub-inhibitory ciprofloxacin pressure might lead to high-level resistance in PA123, with MICs remaining elevated even after drug removal, demonstrating stable resistance and linking sub-MIC exposure to resistance evolution.

**Supplementary Information:**

The online version contains supplementary material available at 10.1007/s11274-026-05105-7.

## Introduction

Fluoroquinolones are commonly used for the treatment of infections caused by *Pseudomonas aeruginosa*, including ocular infections (Wong et al. 2012; McDonald et al. 2014). However, emerging fluoroquinolone resistance worldwide is limiting their efficacy (Shalchi et al. 2011; Willcox 2011; Khan et al. 2020a). Although ophthalmic antibiotics can be applied at concentrations more than 1,000 times higher than those required to inhibit microbial growth in vitro (Chen et al. 2008), the concentration that penetrates the cornea may be less than 10% of what was instilled (Cahane et al. 2004; Kaye et al. 2010). Ciprofloxacin at 3000 µg/mL (0.3%) applied to the cornea achieves aqueous humour levels of only 0.15 ± 0.11 µg/mL (Solomon et al. 2005), which is lower than the minimum inhibitory concentration (MIC) reported for some *P. aeruginosa* isolates (Kaye et al. 2010; Lalitha et al. 2012; Khan et al. 2020b). In addition, the biological activity of an antimicrobial in the cornea may be much lower than its chemical concentration due to interactions with proteins, lipids and other ocular surface components (Kaye et al. 2010). Clinically, higher antibiotic MICs have been associated with delayed healing and poorer outcomes in *P. aeruginosa* corneal infection (Kaye et al. 2010; Lalitha et al. 2012). Therefore, increasing MICs among ocular *P. aeruginosa* isolates may reduce the effective activity of topical fluoroquinolones and highlight the importance of understanding how ciprofloxacin resistance develops.

Resistance in bacteria may be intrinsic, reflecting naturally occurring reduced susceptibility, or acquired through chromosomal mutations or the acquisition of resistance genes (Blair et al. 2015; Munita and Arias, 2016). *P. aeruginosa* can withstand antimicrobial exposure through a range of resistance mechanisms (Poole et al. 1993; Akter et al. 2026). The main mechanisms of fluoroquinolone resistance in *P. aeruginosa* involve mutations in target genes encoding DNA gyrase (*gyrA* and *gyrB*) and topoisomerase IV (*parC* and *parE*) (Akasaka et al. 2001; Lee et al. 2005). In addition, overexpression of RND efflux pumps, including MexAB-OprM, MexEF-OprN, MexXY-OprM and MexCD-OprJ, together with mutations in their regulatory genes, can contribute to reduced fluoroquinolone susceptibility (Jalal et al. 2000; Poole 2001; Zhao et al. 2020; López et al. 2022). Fluoroquinolone resistance may also be associated with acquired quinolone resistance determinants, such as *qnrVC1* and *crpP* which have been reported in *P. aeruginosa* (Khan et al. 2020c, 2021), and are associated with mobile genetic elements, including integrons, plasmids and integrative and conjugative elements (Belotti et al. 2015; Chávez-Jacobo et al. 2018; Botelho et al. 2019; López et al. 2022).

Previous studies on *P. aeruginosa* fluoroquinolone resistance development have focused on laboratory reference strains and clinical strains. In the laboratory reference strain ATCC 27853, exposure to increasing concentrations of ciprofloxacin selected specific quinolone-resistance determining regions (QRDRs) mutations, including *gyrA* Thr83Ile, *gyrA* Asp87Asn, *gyrA* Asp87Gly and *gyrB* Ser466Phe, and identified MexCD-OprJ overexpression as the main efflux-pump mechanism associated with fluoroquinolone resistance (Zhao et al. 2020). A similar study using ciprofloxacin-resistant mutants derived from clinical strains of *P. aeruginosa* showed that ciprofloxacin exposure selected mutants with overexpression of the MexEF-OprN efflux system, with mutations identified in regulatory genes including *mexS*, *mexT* and/or *mvaT* (Llanes et al. 2011). Other studies have reported that different mutations in DNA gyrase and topoisomerase IV, efflux pumps and their regulatory genes of *P. aeruginosa* are associated with fluoroquinolone resistance in ocular infection (Subedi et al. 2018; Akter et al. 2025). However, there is lack of information about the stability of mutations before and after exposure to antibiotic doses, which is a critical unanswered question in ocular infection. This is particularly important because topically administered antibiotics often reach sub-MIC levels in the cornea due to dilution, tear turnover, and variable tissue penetration (Dubald et al. 2018), creating conditions where resistant mutations can arise, and determining whether such mutations persist or revert is essential to understand the potential for long-term resistance development and to guide rational dosing strategies for ocular pathogens. This might be the reason that studies have reported that prior ocular exposure to fluoroquinolones results in more frequent isolation of pathogens with increases in MICs (Fintelmann et al. 2011; Ray et al. 2013).

Therefore, the current study examined the effect of exposing a *P. aeruginosa* ocular isolate to increasing sub-inhibitory concentrations of ciprofloxacin, followed by re-evaluation after ciprofloxacin removal, to identify changes in genes associated with fluoroquinolone resistance.

## Methods

### Growth of *P. aeruginosa* isolates in sub-MIC concentrations of ciprofloxacin

*P. aeruginosa* PA123, an isolate from a corneal infection, was retrieved from − 80 °C stocks of the culture collection of School of Optometry and Vision Science, University of New South Wales, UNSW Sydney, Australia and grown in trypticase soy broth and agar (Sigma-Aldrich, Sydney, Australia) at 37 °C (Khan et al. 2020b). The isolate was collected from a patient without identifiable patient data. PA123 was selected for this study because it was classified as ciprofloxacin-susceptible according to the breakpoint used in the previous study (Akter et al. 2025), which followed CLSI interpretive criteria for *P. aeruginosa* with a baseline ciprofloxacin MIC of 1 µg/mL. Previous whole-genome analysis showed that PA123 lacked canonical QRDR mutations in *gyrA*, *gyrB*, *parC* and *parE*, and did not carry the acquired fluoroquinolone-resistance genes, *crpP* or *qnrVC1* (Akter et al. 2025). PA123 carried baseline SNPs in fluoroquinolone resistance-associated genes, including *parC* Ser485Ala, *nalC* Ser209Arg, *mexT* Pro60Ser, *mexC* Ala328Val, *mexD* Glu257Gln, *oprJ* Asp68Gly/Met69Val and *mexY* Gln840Glu; however, these SNPs were not correlated with fluoroquinolone resistance (Akter et al. 2025) and were interpreted as background polymorphisms rather than established pre-existing resistance mechanisms. Using a genetically susceptible background allowed the specific examination of the stepwise emergence and stability of fluoroquinolone resistance under graded ciprofloxacin exposure, without confounding effects from canonical pre-existing fluoroquinolone resistance mechanisms.

**Fig. 1:**
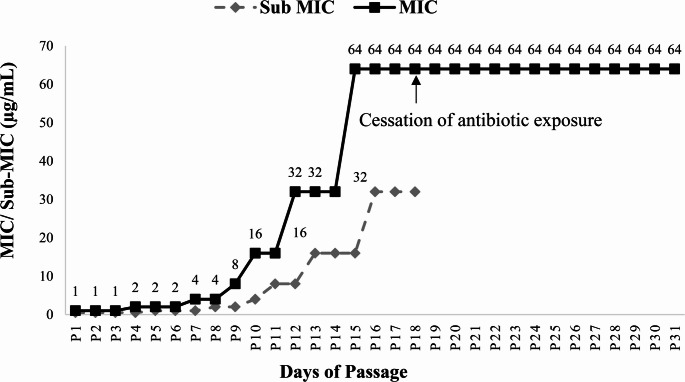
MIC changes of PA123 during exposure to ciprofloxacin over 18 days of growth (P1-P18) and MIC changes after removal of ciprofloxacin from the media (P19-P31). The figure also shows the sub-MIC that the cells were exposed to during growth

At passage P1, PA123 was grown in a sub-inhibitory concentration (sub-MIC) of ciprofloxacin at 0.5 µg/mL at 37 °C for 18 to 24 h. PA123 was then repeatedly exposed to sub-inhibitory concentrations of ciprofloxacin (Sigma-Aldrich, St. Louis, MO, USA) over 18 days (Fig. 1). Each new passage was initiated from the previous broth culture rather than from a single colony. Briefly, bacteria from the previous passage were used to prepare a standardised inoculum of 5 × 10⁵ CFU/mL, which was then inoculated into fresh medium containing the next sub-inhibitory concentration of ciprofloxacin. Therefore, the experiment followed an evolving bacterial population across passages rather than a single colony-derived lineage. The MIC of the isolate to ciprofloxacin was assayed each day after exposure to the antibiotic in cation-adjusted Mueller-Hinton broth (MHB; Oxoid Ltd.) using the broth microdilution method (Akter et al. 2025) and changes to its MIC was used to change the subsequent sub-inhibitory concentrations in passages (Fig. 1). Bacterial cells from each passage were collected, and aliquots were stored at -80 °C in 30% glycerol for subsequent extraction of DNA for whole genome sequencing (WGS). After 18 passages, ciprofloxacin exposure was withdrawn, and the strain was further passaged in antibiotic-free medium for 13 days to assess the persistence of resistance in the absence of selective pressure. A control culture was sub-cultured every day in antibiotic-free medium for 31 days.

### Whole genome sequencing and mutations analysis

WGS was performed on genomic DNA extracted from isolates collected from selected passages (P1, P4, P7, P9, P10, P12, P15, and P18) based on defined changes in ciprofloxacin susceptibility. P1, P4, P7, P9, P10, P12 and P15 passages were chosen because each represented a point at which MIC doubled compared to the preceding level, thereby capturing discrete evolutionary steps during resistance development under sub-MIC ciprofloxacin exposure. Passage P18 was selected because, although resistance first reached a high level at P15 (MIC = 64 µg/mL), the MIC remained stable and unchanged from P15 through P18, indicating a plateau phase of resistance. Sequencing P18 therefore allowed assessment of whether additional genetic changes accumulated after resistance stabilization despite continued antibiotic exposure.

To assess the stability of ciprofloxacin resistance in the absence of antibiotic pressure, WGS was additionally performed on passages carried out without ciprofloxacin (P19, P26, and P31). These passages were selected to represent early (P19), intermediate (P26), and late (P31) stages following antibiotic withdrawal, allowing evaluation of whether resistance-associated mutations were maintained, reverted, or further evolved over time. In parallel, control lineages passaged without antibiotic exposure were sequenced at P18 and P31 to distinguish ciprofloxacin-driven genetic changes from background mutations arising due to serial passaging. This comparison ensured that observed mutations in the ciprofloxacin-exposed lineage were attributable to antibiotic selection rather than laboratory adaptation.

For WGS, genomic DNA was extracted from cells collected from the above-mentioned selected passages using DNeasy Blood and Tissue Kit (Qiagen, Hilden Germany) according to the manufacturer’s instructions. DNA concentration and integrity were evaluated using a Qubit 3.0 fluorometer (Thermo Fisher Scientific, Wilmington, USA) and 1% agarose gel electrophoresis (Tanon, Shanghai, China). Libraries prepared with the VAHTS Universal Plus DNA Library Prep Kit V4 (ND801, Vazyme, Nanjing, China) were quality-checked using a QSep-400 system (BiOptic, Taipei, China) and sequenced on the Illumina NovaSeq X platform (Illumina, San Diego, USA) with paired-end 150 bp reads. The raw sequencing reads generated in this study have been deposited in the NCBI BioProject database under accession number PRJNA1376985. The quality of raw sequencing data (.fastq files) was assessed using FastQC v0.11.9 (Andrews 2010), confirming high-quality reads (Phred > 30), and subsequently processed with Trimmomatic v0.38 (Bolger et al. 2014) to trim low-quality bases and remove adapter sequences. The trimmed reads were subjected to analysis using Snippy 4.6.0 (Seemann 2015) for detection of single nucleotide polymorphisms (SNPs) using the parent strain PA123 as a reference retrieved from NCBI (BioProject number: PRJNA590804) (Khan et al. 2020a). For *gyrA* SNPs resulting in amino acid substitutions, the predicted functional effect was assessed using the Sorting Intolerant From Tolerant (SIFT) tool (Ng and Henikoff, 2003). Substitutions with a SIFT score ≤ 0.05 were considered deleterious.

## Results

The parent strain PA123 had an initial MIC of 1 µg/mL (Akter et al. 2025), which increased gradually with continued exposure of the ciprofloxacin (Fig. 1). A two-fold increase in MIC was first observed at passage 4 (2 µg/mL), followed by sequential increases at passage 7 (4 µg/mL), passage 9 (8 µg/mL), passage 10 (16 µg/mL), and passage 12 (32 µg/mL) (Fig. 1), indicating stepwise adaptation under sub-MIC pressure. At passage 15, the MIC further jumped 2-fold to 64 µg/mL which represents a 64-fold increase compared to the parent strain. After the 18 passage, ciprofloxacin exposure was stopped and in the absence of antibiotic pressure, the elevated MIC of 64 µg/mL persisted through passages 19 to 31.

Analysis of WGS of selected passages revealed a stepwise accumulation of mutations associated with ciprofloxacin resistance (Table 1; all acquired mutations are presented in Supplementary Table 1). Early passages (P1-P7) with MIC ranging from 1 to 4 µg/mL carried a consistent frameshift mutation Leu240fs (fs = frame shift) in *mexS*, an oxidoreductase that negatively regulates the MexEF-OprN efflux pump (Morita et al. 2015). At passage P4, a mutation at amino acid position 29 resulted in a substitution of valine to glycine in the GNQ09_02850 gene which encodes Sigma-70 family RNA polymerase sigma factor; however, this SNP disappeared after this passage.

**Table 1:** Mutations associated with ciprofloxacin resistance acquired by PA123 during passages with and without exposure of ciprofloxacin

Passage	MIC	Sub-MIC	Effect	Gene	Product	Mutation status
Growth in sub-MIC ciprofloxacin						
P1	1	0.5	Leu240fs	*mexS*	Oxidoreductase MexS	✦ Appeared at P1; maintained through P31
P4	2	0.5	Val29Gly	GNQ09_02850	Sigma-70 family RNA polymerase sigma factor	✧ Appeared at P4; not maintained (absent from P7 onwards)
			Leu240fs	*mexS*	Oxidoreductase MexS	✦ Maintained
P7	4	1	Leu240fs	*mexS*	Oxidoreductase MexS	✦ Maintained
P9	8	2	Arg342His	GNQ09_09635	EAL domain-containing protein	✦ Appeared at P9; maintained through P31
			Thr83Ile	*gyrA*	DNA gyrase subunit A	✧ Appeared at P9; not maintained (absent from P10 onwards)
			Leu240fs	*mexS*	Oxidoreductase MexS	✦ Maintained
P10	16	4	Arg342His	GNQ09_09635	EAL domain-containing protein	✦ Maintained
			Ala51Val	*gyrA*	DNA gyrase subunit A	✧ Appeared at P10; not maintained (absent from P12 onwards)
			Leu240fs	*mexS*	Oxidoreductase MexS	✦ Maintained
P12	32	8	Arg342His	GNQ09_09635	EAL domain-containing protein	✦ Maintained
			Asp87Tyr	*gyrA*	DNA gyrase subunit A	✦ Appeared at P12; maintained through P31
			Ala570dup	*gyrA*	DNA gyrase subunit A	✦ Appeared at P12; maintained through P31
			Leu240fs	*mexS*	Oxidoreductase MexS	✦ Maintained
P15	64	16	His137fs	GNQ09_05810	Hypothetical protein	✦ Appeared at P15; maintained through P31
			Arg342His	GNQ09_09635	EAL domain-containing protein	✦ Maintained
			Asp87Tyr	*gyrA*	DNA gyrase subunit A	✦ Maintained
			Ala570dup	*gyrA*	DNA gyrase subunit A	✦ Maintained
			Leu240fs	*mexS*	Oxidoreductase MexS	✦ Maintained
P18	64	32	His137fs	GNQ09_05810	Hypothetical protein	✦ Maintained
			Arg342His	GNQ09_09635	EAL domain-containing protein	✦ Maintained
			Asp87Tyr	*gyrA*	DNA gyrase subunit A	✦ Maintained
			Ala570dup	*gyrA*	DNA gyrase subunit A	✦ Maintained
			Leu240fs	*mexS*	Oxidoreductase MexS	✦ Maintained
Growth in absence of any ciprofloxacin						
P19	64	0	His137fs	GNQ09_05810	Hypothetical protein	✦ Maintained
			Arg342His	GNQ09_09635	EAL domain-containing protein	✦ Maintained
			Asp87Tyr	*gyrA*	DNA gyrase subunit A	✦ Maintained
			Ala570dup	*gyrA*	DNA gyrase subunit A	✦ Maintained
			Leu240fs	*mexS*	Oxidoreductase MexS	✦ Maintained
P26	64		His137fs	GNQ09_05810	Hypothetical protein	✦ Maintained
			Arg342His	GNQ09_09635	EAL domain-containing protein	✦ Maintained
			Asp87Tyr	*gyrA*	DNA gyrase subunit A	✦ Maintained
			Ala570dup	*gyrA*	DNA gyrase subunit A	✦ Maintained
			Leu240fs	*mexS*	Oxidoreductase MexS	✦ Maintained
P31	64		His137fs	GNQ09_05810	Hypothetical protein	✦ Maintained
			Arg342His	GNQ09_09635	EAL domain-containing protein	✦ Maintained
			Asp87Tyr	*gyrA*	DNA gyrase subunit A	✦ Maintained
			Ala570dup	*gyrA*	DNA gyrase subunit A	✦ Maintained
			Leu240fs	*mexS*	Oxidoreductase MexS	✦ Maintained
Changes to PA123 during growth without ciprofloxacin (control)						
P18C	1	0	Pro270Gln	*tagH*	Type VI secretion system-associated FHA domain protein TagH	Background mutation; not resistance-related
P31C			Pro270Gln	*tagH*	Type VI secretion system-associated FHA domain protein TagH	Background mutation; not resistance-related
			Arg48Pro	GNQ09_25310	DUF692 family protein	Background mutation; not resistance-related

*fs* frameshift mutation, *dup* duplication, ✦ mutation appeared and was maintained in all subsequent passages;✧ mutation appeared transiently and was not maintained in subsequent passages; *P* ciprofloxacin-exposed passage, *C* antibiotic-free control passage

With increases in resistance to 8 µg/mL, along with *mexS* mutation, mutations accumulated in the *gyrA* gene, a primary target of fluoroquinolones (Akasaka et al. 2001; Lee et al. 2005; Nouri et al. 2016). At passages P9 (MIC 8 µg/mL) and P10 (MIC 16 µg/mL), PA123 acquired Thr83Ile mutation and a novel functional mutation Ala51Val, respectively, which disappeared in P12 (MIC 32 µg/mL). At passage 12, PA123 acquired Asp87Tyr and an alanine duplication at position 570 (Ala570dup) in *gyrA* and these mutations remained unchanged when the MIC doubled (64 µg/mL) at passage 15 and continued to be present up to passage 18 (MIC 64 µg/mL).

From passage 15 (MIC 64 µg/mL), a frameshift mutation originating at histidine position 137 was detected in a hypothetical protein encoded from GNQ09_05810, located between the *parC* and *parE* genes, and it persisted through passage 31. This mutation was associated with the highest level of ciprofloxacin resistance, as it was specifically found in isolates with an MIC of 64 µg/mL. An additional novel Arg342His mutation appeared in GNQ09_09635 gene which encodes an EAL domain-containing protein (Arg342His) from passages P9 (MIC 8 µg/mL) to P31 (MIC 64 µg/mL), possibly affecting cyclic di-guanosine monophosphate (cyclic-di-GMP) signalling and stress adaptation, although its significance to ciprofloxacin resistance in unknown.

When the antibiotic selection pressure was removed, PA123 remained highly resistant to ciprofloxacin (64 µg/mL) for another 13 days of passage. This was associated with maintenance of the SNPs in His137fs (GNQ09_05810), Arg342His (GNQ09_09635), Asp87Tyr (*gyrA*), Ala570dup (*gyrA*), and Leu240fs (*mexS*). In contrast, PA123 without antibiotic exposure at the control passages P18C and P31C showed only background mutations (*tagH* Pro270Gln, GNQ09_25310 Arg48Pro) unrelated to ciprofloxacin resistance (Subedi et al. 2018; Akter et al. 2025).

## Discussion

Repeated exposure of PA123 to ciprofloxacin for 18 passages led to an increased MIC. This observed change in *P. aeruginosa* may have been driven by exposure to ciprofloxacin at varying or intermittent concentrations due to consumption during growth (Gould and MacKenzie, 2002). These findings suggested that survival in antimicrobials was an adaptable phenomenon (Martinez 2017). This situation is alarming as increasing fluoroquinolone resistance results in an increased treatment cost due to prolonged healing of eye infections where fluoroquinolones are considered as a first line empirical treatment (Gokhale 2008).

The MIC of PA123 climbed from 1 to 64 µg/mL by passage 15, a 64-fold increase, and remained at this concentration thereafter. WGS data revealed a chronological sequence of adaptations: first a novel frameshift mutation in *mexS* (Leu240fs), then classic quinolone target mutations in *gyrA* (Thr83Ile, Ala51Val, Asp87Tyr, and Ala570dup). Additional Arg342His change occurred in EAL-domain phosphodiesterase and finally a frameshift mutation His137fs in a hypothetical gene located between topoisomerase IV subunits A and B. No such mutations were present in antibiotic-free controls, underscoring that serial sub-MIC exposure specifically selected this trajectory. This sequential pattern of initial efflux activation followed by topoisomerase mutations explains the stepwise MIC jump that was observed (from single-digit to double-digit µg/mL), mirroring the way *P. aeruginosa* can accumulate resistance alleles under sustained stress (Yu et al. 2025).

The mutational cascade found aligns closely with established fluoroquinolone resistance paradigms in *P. aeruginosa*. Ciprofloxacin resistance is typically multifactorial, involving both drug-target alterations and efflux pump activation (Jalal et al. 2000; Poole 2001; Khan et al. 2020a, 2021; López et al. 2022). In particular, *mexS* inactivation is known to upregulate the MexEF-OprN efflux system (Morita et al. 2015; Xu et al. 2021). One study demonstrated that introducing a MexS Phe7Ser mutation, which disrupts MexS function, led to MexEF-OprN hyperexpression and an 8-fold increase in ciprofloxacin MIC (Xu et al. 2021). Another study reported additional MexS substitutions in clinical *nfxC* mutants of *P. aeruginosa*, including Asp44Glu, Ser60Phe, Ser60Pro, Val73Ala, Val104Ala, Ala166Pro, Phe185Leu, Cys245Gly, Phe253Leu, Leu263Gln and Leu270Gln, which were associated with altered MexS function and MexEF-OprN overproduction (Richardot et al. 2016). Early *mexS* frameshift in present study may be associated with hyper-expressed MexEF-OprN, potentially conferring an initial survival advantage under low ciprofloxacin and priming the bacterium for the later strong resistance from *gyrA* changes.

Subsequent *gyrA* mutations then raised the MIC further, similar to a previous experimental study with engineered *P. aeruginosa* strains which showed that *gyrA* (especially at codon 83) is the critical first step for significant ciprofloxacin resistance (Rehman et al. 2021). In PA123, a threonine to isoleucine mutation at position 83 of *gyrA* was present on passage 9 (MIC 8 µg/mL). A recent study used similar methods to the present study for developing resistant strains in a standard laboratory strain of *P. aeruginosa* (ATCC 27853) and also reported the Thr83Ile mutation in the *gyrA* gene (Zhao et al. 2020). Thr83Ile mutation remained present from passage 3 (2 MIC) to passage 8 (64 MIC) but disappeared thereafter and a novel functional mutation Ala51Val in the *gyrA* was detected from passage 10 (Supplementary Table 2). The Thr83Ile has been previously reported in whole genome analyses of ciprofloxacin resistant strains isolated from keratitis, burns and other infections (Yonezawa et al. 1995; Lomholt and Kilian, 2003; Farahi et al. 2018). The disappearance of the Ala51Val substitution suggests that this mutation may contribute to the initial increase in MIC, but its precise role requires further investigation.

Asp87Tyr and Ala570dup (a novel mutation first described in this study, Supplementary Table 2) mutations were detected on passage 12 and remained unchanged to the last passage. Mutations at 87 position of *gyrA* have been reported in a previous evolution study but instead of Asp87Tyr that previous study reported Asp87Asn which was associated with increased MIC (Zhao et al. 2020). The Asp87Tyr SNP has been reported previously in *P. aeruginosa* isolates from other infections, and whilst it is not always associated with high increases in MIC to fluoroquinolones, it can be especially if combined with other SNPs (Lee et al. 2005; Matsumoto et al. 2012; Park et al. 2020). The Ala570dup SNP appears to be a novel mutation event. This SNP inserts an additional alanine amino acid at this position in the protein. The effect of this mutation on the function of GyrA is not yet known.

In this evolution experiment, the Thr83Ile and Asp87Tyr changes in GyrA are canonical resistance alleles and both were consistent with previous studies (Bruchmann et al. 2013; Wang et al. 2014; Yang et al. 2015; Akter et al. 2025). The combined and stable effects of mutations in GyrA (Asp87Tyr and Ala570dup) and MexS (Leu240fs) might have association with high level of MIC, though the roles of these new mutations (Ala570dup in GyrA and Leu240fs in MexS) need to be elucidated. These results recapitulate this hierarchy: an efflux mutation gave an early boost, then target-site mutations along with efflux pumps mutations resulted in full resistance (Rehman et al. 2021; Xu et al. 2021).

The Arg342His mutation in GNQ09_09635 which encodes an EAL-domain phosphodiesterase might suggest selection on cyclic-di-GMP signalling or biofilm regulation. EAL proteins degrade cyclic-di-GMP, so this mutation might be associated with elevated cyclic-di-GMP levels (Valentini and Filloux, 2016). High cyclic-di-GMP is linked to biofilm-like states and general drug tolerance. For instance, small-colony variants with elevated cyclic-di-GMP are known to be more resistant to antibiotics (Valentini and Filloux, 2016). Thus, the EAL-domain change may reflect a collateral adaptation, perhaps promoting a sessile phenotype or stress response that complements efflux and target mutations. Another novel frameshift mutation His137fs in the hypothetical gene-GNQ09_05810 might have association in resistance development through changing the target site as located between topoisomerase subunits encoding genes *parC* and *parE*. Importantly, these two mutations remained fixed even after ciprofloxacin was removed, indicating possible correlation with resistance.

Overall findings indicated that sub-MIC ciprofloxacin exposure can be a potent driver of resistance and effectively pushed PA123 along a well-known mutational route (efflux → gyrase) to high-level resistance. This is consistent with the previous study that showed that strains may become resistant if exposed to increasing antibiotic concentration for longer periods due to the development of more stable gene mutations (Windels et al. 2019). This study, along with a previous study examining the ability of *P. aeruginosa* to become tolerant to ciprofloxacin (Khan et al. 2023), underscores the need to apply high doses of drugs such as ciprofloxacin to the eye during infection to reduce the development of resistance and the possible re-emergence of the infection when antibiotics are stopped.

This study has several limitations. First, additional phenotypic assays were not performed in the present study. In particular, growth curves were not generated for the parental PA123 isolate, the ciprofloxacin-resistant derivatives or the antibiotic-free passage controls. Therefore, it cannot be determined whether the acquisition of stable ciprofloxacin resistance was associated with a measurable growth fitness cost under antibiotic-free conditions. Future studies should compare the growth kinetics of PA123, ciprofloxacin-resistant derivatives and antibiotic-free passage controls to determine whether the resistance trajectory observed here alters bacterial fitness.

A further limitation of this study is that susceptibility testing was restricted to ciprofloxacin. Therefore, it remains unknown whether the *mexS* Leu240fs-carrying passaged PA123 derivatives showed collateral changes in susceptibility to other clinically relevant topical antimicrobials for ocular infection. Based on previous studies, *mexS* alterations may affect MexEF-OprN regulation and have been associated with reduced fluoroquinolone susceptibility, including ciprofloxacin resistance (Llanes et al. 2011; Morita et al. 2015; Xu et al. 2021), making altered susceptibility to other fluoroquinolones biologically plausible. However, cross-resistance to antibiotics other than ciprofloxacin cannot be inferred from the present data. Additional MIC testing, together with functional validation of the *mexS* mutation and other candidate mutations, would be required to determine whether such collateral susceptibility changes occurred.

Moreover, in this study, genomic DNA was extracted from bacteria collected from liquid broth cultures at selected passages, representing the dominant genomic content of the evolving bacterial population at each sampled time point. This approach allowed detection of the major resistance-associated mutations that became established during ciprofloxacin exposure, but it may not capture the full spectrum of within-population genotypic heterogeneity. Individual cells or sub-populations within the same passage may have acquired additional transient or low-frequency mutations that were not detected by this sequencing approach. This may partly explain why relatively few mutations were observed outside the main fluoroquinolone resistance-associated changes. Future studies using single-colony sequencing or deep population sequencing across additional passages would provide a more comprehensive view of within-population diversity and parallel mutational trajectories under sub-inhibitory ciprofloxacin exposure.

## Conclusion

Growth of PA123 in the presence of increasing sub-inhibitory concentrations of ciprofloxacin imposed selective pressure associated with the emergence of mutations in fluoroquinolone resistance-related genes. Overall, the emergence of mutations in *gyrA* and *mexS* is consistent with known ciprofloxacin resistance pathways involving target-site modification and efflux regulation. However, the level of supporting evidence differed between individual mutations: Thr83Ile and Asp87Tyr in *gyrA* are well documented in fluoroquinolone resistance, whereas Ala51Val and Ala570dup are uncharacterised in this context and require further functional validation (Supplementary Table 2). Although the specific *mexS* Leu240fs mutation has not been previously described, loss-of-function alterations in *mexS* are consistent with MexEF-OprN overproduction and reduced fluoroquinolone susceptibility. 

## Supplementary Information

Below is the link to the electronic supplementary material.

Supplementary Material 1 (DOCX 22.7 KB)

Supplementary Material 2 (DOCX 41.9 KB)

## Data Availability

Data is provided within the manuscript or supplementary information files. The sequencing data generated in this study have been submitted to the NCBI Sequence Read Archive (SRA) under BioProject ID PRJNA1376985.
