# Supplementary material for: Effects of repeated culture in sub-inhibitory concentrations of ciprofloxacin on resistance and genetic characteristics of an ocular Pseudomonas aeruginosa isolate
Source: World J Microbiol Biotechnol. 2026 Jun 29;42(7):377. doi: 10.1007/s11274-026-05105-7 (PMC13314826; doi:10.1007/s11274-026-05105-7)
Supplement: Supplementary file 1 — Supplementary Material 1 (DOCX 22.7 KB) [file 11274_2026_5105_MOESM1_ESM.docx]

Supplementary Table 1: All mutations acquired by PA123 during passages with and without exposure of ciprofloxacin

| Passage | MIC | LOCUS_TAG | Effect | Gene | Product | Mutation Status |
| --- | --- | --- | --- | --- | --- | --- |
| P1 | 1 | GNQ09_08635 | Pro270Gln | *tagH* | Type VI secretion system-associated FHA domain protein TagH | Background mutation; not resistance-related |
|  |  | GNQ09_17345 | Leu240fs | *mexS* | Oxidoreductase MexS | ✦ Appeared at P1; maintained through P31 |
| P4 | 2 | GNQ09_02850 | Val29Gly | GNQ09_02850 | Sigma-70 family RNA polymerase sigma factor | ✧ Appeared at P4; not maintained (absent from P7 onwards) |
|  |  | GNQ09_08635 | Pro270Gln | *tagH* | Type VI secretion system-associated FHA domain protein TagH | Background mutation; not resistance-related |
|  |  | GNQ09_17345 | Leu240fs | *mexS* | Oxidoreductase MexS | ✦ Maintained |
| P7 | 4 | GNQ09_08635 | Pro270Gln | *tagH* | Type VI secretion system-associated FHA domain protein TagH | Background mutation; not resistance-related |
|  |  | GNQ09_17345 | Leu240fs | *mexS* | Oxidoreductase MexS | ✦ Maintained |
| P9 | 8 | GNQ09_08635 | Pro270Gln | *tagH* | Type VI secretion system-associated FHA domain protein TagH | Background mutation; not resistance-related |
|  |  | GNQ09_09635 | Arg342His | GNQ09_09635 | EAL domain-containing protein | ✦ Appeared at P9; maintained through P31 |
|  |  | GNQ09_09785 | Thr83Ile | *gyrA* | DNA gyrase subunit A | ✧ Appeared at P9; not maintained (absent from P10 onwards) |
|  |  | GNQ09_17345 | Leu240fs | *mexS* | Oxidoreductase MexS | ✦ Maintained |
| P10 | 16 | GNQ09_08635 | Pro270Gln | *tagH* | Type VI secretion system-associated FHA domain protein TagH | Background mutation; not resistance-related |
|  |  | GNQ09_09635 | Arg342His | GNQ09_09635 | EAL domain-containing protein | ✦ Maintained |
|  |  | GNQ09_09785 | Ala51Val | *gyrA* | DNA gyrase subunit A | ✧ Appeared at P10; not maintained (absent from P12 onwards) |
|  |  | GNQ09_17345 | Leu240fs | *mexS* | Oxidoreductase MexS | ✦ Maintained |
| P12 | 32 | GNQ09_08635 | Pro270Gln | *tagH* | Type VI secretion system-associated FHA domain protein TagH | Background mutation; not resistance-related |
|  |  | GNQ09_09635 | Arg342His | GNQ09_09635 | EAL domain-containing protein | ✦ Maintained |
|  |  | GNQ09_09785 | Asp87Tyr | *gyrA* | DNA gyrase subunit A | ✦ Appeared at P12; maintained through P31 |
|  |  | GNQ09_09785 | Ala570dup | *gyrA* | DNA gyrase subunit A | ✦ Appeared at P12; maintained through P31 |
|  |  | GNQ09_17345 | Leu240fs | *mexS* | Oxidoreductase MexS | ✦ Maintained |
| P15 | 64 | GNQ09_05810 | His137fs | GNQ09_05810 | Hypothetical protein | ✦ Appeared at P15; maintained through P31 |
|  |  | GNQ09_08635 | Pro270Gln | *tagH* | Type VI secretion system-associated FHA domain protein TagH | Background mutation; not resistance-related |
|  |  | GNQ09_09635 | Arg342His | GNQ09_09635 | EAL domain-containing protein | ✦ Maintained |
|  |  | GNQ09_09785 | Asp87Tyr | *gyrA* | DNA gyrase subunit A | ✦ Maintained |
|  |  | GNQ09_09785 | Ala570dup | *gyrA* | DNA gyrase subunit A | ✦ Maintained |
|  |  | GNQ09_17345 | Leu240fs | *mexS* | Oxidoreductase MexS | ✦ Maintained |
| P18 | 64 | GNQ09_05810 | His137fs | GNQ09_05810 | Hypothetical protein | ✦ Maintained |
|  |  | GNQ09_08635 | Pro270Gln | *tagH* | Type VI secretion system-associated FHA domain protein TagH | Background mutation; not resistance-related |
|  |  | GNQ09_09635 | Arg342His | GNQ09_09635 | EAL domain-containing protein | ✦ Maintained |
|  |  | GNQ09_09785 | Asp87Tyr | *gyrA* | DNA gyrase subunit A | ✦ Maintained |
|  |  | GNQ09_09785 | Ala570dup | *gyrA* | DNA gyrase subunit A | ✦ Maintained |
|  |  | GNQ09_17345 | Leu240fs | *mexS* | Oxidoreductase MexS | ✦ Maintained |
| P19 | 64 | GNQ09_05810 | His137fs | GNQ09_05810 | Hypothetical protein | ✦ Maintained |
|  |  | GNQ09_08635 | Pro270Gln | *tagH* | Type VI secretion system-associated FHA domain protein TagH | Background mutation; not resistance-related |
|  |  | GNQ09_09635 | Arg342His | GNQ09_09635 | EAL domain-containing protein | ✦ Maintained |
|  |  | GNQ09_09785 | Asp87Tyr | *gyrA* | DNA gyrase subunit A | ✦ Maintained |
|  |  | GNQ09_09785 | Ala570dup | *gyrA* | DNA gyrase subunit A | ✦ Maintained |
|  |  | GNQ09_17345 | Leu240fs | *mexS* | Oxidoreductase MexS | ✦ Maintained |
| P26 | 64 | GNQ09_05810 | His137fs | GNQ09_05810 | Hypothetical protein | ✦ Maintained |
|  |  | GNQ09_08635 | Pro270Gln | *tagH* | Type VI secretion system-associated FHA domain protein TagH | Background mutation; not resistance-related |
|  |  | GNQ09_09635 | Arg342His | GNQ09_09635 | EAL domain-containing protein | ✦ Maintained |
|  |  | GNQ09_09785 | Asp87Tyr | *gyrA* | DNA gyrase subunit A | ✦ Maintained |
|  |  | GNQ09_09785 | Ala570dup | *gyrA* | DNA gyrase subunit A | ✦ Maintained |
|  |  | GNQ09_17345 | Leu240fs | *mexS* | Oxidoreductase MexS | ✦ Maintained |
| P31 | 64 | GNQ09_05810 | His137fs | GNQ09_05810 | Hypothetical protein | ✦ Maintained |
|  |  | GNQ09_08635 | Pro270Gln | *tagH* | Type VI secretion system-associated FHA domain protein TagH | Background mutation; not resistance-related |
|  |  | GNQ09_09635 | Arg342His | GNQ09_09635 | EAL domain-containing protein | ✦ Maintained |
|  |  | GNQ09_09785 | Asp87Tyr | *gyrA* | DNA gyrase subunit A | ✦ Maintained |
|  |  | GNQ09_09785 | Ala570dup | *gyrA* | DNA gyrase subunit A | ✦ Maintained |
|  |  | GNQ09_17345 | Leu240fs | *mexS* | Oxidoreductase MexS | ✦ Maintained |
| P18C | 1 | GNQ09_08635 | Pro270Gln | *tagH* | Type VI secretion system-associated FHA domain protein TagH | Background mutation; not resistance-related |
| P31C | 1 | GNQ09_08635 | Pro270Gln | *tagH* | Type VI secretion system-associated FHA domain protein TagH | Background mutation; not resistance-related |
|  |  | GNQ09_25310 | Arg48Pro | GNQ09_25310 | DUF692 family protein | Background mutation; not resistance-related |

**fs = frameshift mutation; dup = duplication**

**✦ mutation appeared and was maintained in all subsequent passages.**

**✧ mutation appeared transiently and was not maintained in subsequent passages.**

**P, ciprofloxacin-exposed passage; C, antibiotic-free control passage.**
