# Supplementary material for: Effects of repeated culture in sub-inhibitory concentrations of ciprofloxacin on resistance and genetic characteristics of an ocular Pseudomonas aeruginosa isolate
Source: World J Microbiol Biotechnol. 2026 Jun 29;42(7):377. doi: 10.1007/s11274-026-05105-7 (PMC13314826; doi:10.1007/s11274-026-05105-7)
Supplement: Supplementary file 2 — Supplementary Material 2 (DOCX 41.9 KB) [file 11274_2026_5105_MOESM2_ESM.docx]

Supplementary Table 2. Literature support and predicted implication of *gyrA* alterations detected during ciprofloxacin exposure of PA123.

| GyrA alteration | Literature support / SIFT prediction | Implication in fluoroquinolone resistance |
| --- | --- | --- |
| Thr83Ile | Previously reported in fluoroquinolone-resistant *P. aeruginosa* and recognized as a common quinolone-resistance-determining region (QRDR)-associated *gyrA* substitution ([Yonezawa et al., 1995](#_ENREF_7); [Akasaka et al., 2001](#_ENREF_1); [Lee et al., 2005](#_ENREF_3); [Matsumoto et al., 2012](#_ENREF_4); [Sada et al., 2022](#_ENREF_6)). SIFT predicted this substitution to be deleterious. | This substitution is likely to contribute to reduced fluoroquinolone susceptibility through target alteration of DNA gyrase. High-level fluoroquinolone resistance is more commonly associated with the accumulation of additional QRDR mutations, such as further GyrA substitutions at residue 87 and/or mutations in ParC/ParE ([Nouri et al., 2016](#_ENREF_5)) |
| Asp87Tyr | Previously reported in *P. aeruginosa* clinical isolates with fluoroquinolone resistance. Mutations at GyrA residue 87, including Asp87Tyr and Asp87Asn, are recognized QRDR-associated GyrA substitutions ([Matsumoto et al., 2012](#_ENREF_4); [Sada et al., 2022](#_ENREF_6)). SIFT predicted this substitution to be deleterious. | Asp87Tyr has been detected in fluoroquinolone-resistant clinical *P. aeruginosa* isolates, often in combination with Thr83Ile. Substitutions at GyrA residue Asp87, including Asp87Asn and Asp87Gly, together with additional QRDR mutations in *gyrA* and/or *parC*, have been associated with high-level fluoroquinolone resistance by altering fluoroquinolone-target interactions ([Yonezawa et al., 1995](#_ENREF_7); [Matsumoto et al., 2012](#_ENREF_4)). |
| Ala51Val | Not identified as an established fluoroquinolone-resistance mutation in *P. aeruginosa* in the available literature. SIFT predicted this substitution to be deleterious. | This mutation appeared transiently at P10 (MIC 16 µg/mL) and was absent by P12, suggesting it may represent an intermediate evolutionary step rather than a stable resistance determinant. The predicted deleterious effect suggests possible alteration of GyrA protein function, but its specific contribution to fluoroquinolone resistance remains uncertain and requires functional validation. |
| Ala570dup | Not identified as a previously documented fluoroquinolone-resistance mutation in *P. aeruginosa* in the available literature. This is an in-frame alanine duplication and therefore alters the predicted GyrA protein sequence. | This mutation emerged at P12 alongside Asp87Tyr (MIC 32 µg/mL) and persisted through P18 and all post-withdrawal passages (P19-P31), during which stable high-level resistance was observed (MIC 64 µg/mL). This suggests it may contribute to resistance maintenance, although direct functional validation is required to confirm its role. |

***SIFT= Sorting Intolerant From Tolerant; dup=amino acid duplication.***

**References:**

Akasaka T, Tanaka M, Yamaguchi A, et al. (2001) Type II topoisomerase mutations in fluoroquinolone-resistant clinical strains of *Pseudomonas aeruginosa* isolated in 1998 and 1999: role of target enzyme in mechanism of fluoroquinolone resistance. Antimicrob Agents Chemother 45:2263-2268. <https://doi.org/10.1128/aac.45.8.2263-2268.2001>

Lee JK, Lee YS, Park YK, et al. (2005) Alterations in the GyrA and GyrB subunits of topoisomerase II and the ParC and ParE subunits of topoisomerase IV in ciprofloxacin-resistant clinical isolates of *Pseudomonas aeruginosa.* Int J Antimicrob Agents 25:290-295. <https://doi.org/10.1016/j.ijantimicag.2004.11.012>

Matsumoto M, Shigemura K, Shirakawa T, et al. (2012) Mutations in the *gyrA* and *parC* genes and *in vitro* activities of fluoroquinolones in 114 clinical isolates of *Pseudomonas aeruginosa* derived from urinary tract infections and their rapid detection by denaturing high-performance liquid chromatography. Int J Antimicrob Agents 40:440-444. <https://doi.org/10.1016/j.ijantimicag.2012.06.021>

Nouri R, Ahangarzadeh Rezaee M, Hasani A, et al. (2016) The role of *gyrA* and *parC* mutations in fluoroquinolones-resistant *Pseudomonas aeruginosa* isolates from Iran. Braz J Microbiol 47:925-930. <https://doi.org/10.1016/j.bjm.2016.07.016>

Sada M, Kimura H, Nagasawa N, et al. (2022) Molecular evolution of the *Pseudomonas aeruginosa* DNA gyrase *gyrA* gene. Microorganisms 10: 1660. <https://doi.org/10.3390/microorganisms10081660>

Yonezawa M, Takahata M, Matsubara N, et al. (1995) DNA gyrase *gyrA* mutations in quinolone-resistant clinical isolates of *Pseudomonas aeruginosa*. Antimicrob Agents Chemother 39:1970-1972. <https://doi.org/10.1128/AAC.39.9.1970>
